# Anti-Inflammatory Effect of a Polysaccharide Derived from *Artocarpus heterophyllus* Lam. Pulp on Lipopolysaccharide-Stimulated RAW264.7 Macrophages Through Inhibiting MAPK/ERK Signaling Pathway

**DOI:** 10.3390/nu17243879

**Published:** 2025-12-12

**Authors:** Benyan Bai, Mengyang Liu, Panjie Xu, Yanjun Zhang, Fei Xu, Gang Wu, Yan Zhou, Kexue Zhu

**Affiliations:** 1 The Key Laboratory of Environmental Pollution Monitoring and Disease Control, School of Public Health, Ministry of Education, Guizhou Medical University, Guiyang 561113, China; 2 Spice and Beverage Research Institute, Chinese Academy of Tropical Agricultural Sciences, Wanning 571533, China; 3 Key Laboratory of Processing Suitability and Quality Control of the Special Tropical Crops of Hainan Province, Wanning 571533, China; 4 National Center of Important Tropical Crops Engineering and Technology Research, Wanning 571533, China

**Keywords:** polysaccharide from *Artocarpus heterophyllus* Lam., anti-inflammatory, MAPKs/ERK signaling pathways

## Abstract

Background: Inflammation is a critical pathological process implicated in numerous diseases. Methods: In this study, a water-soluble polysaccharide was extracted from the fruit pulp of *Artocarpus heterophyllus* Lam. (namely, JFP-Ps), and the anti-inflammatory properties and underlying mechanisms were investigated. Inflammatory responses were induced in RAW264.7 macrophages through lipopolysaccharide (LPS) stimulation. Results: JFP-Ps markedly diminished the production of nitric oxide (NO) and reactive oxygen species (ROS); reduced LPS-induced cell apoptosis by increasing glutathione peroxidase (GSH-Px) and superoxide dismutase (SOD) activity; and decreased pro-inflammatory cytokine levels, including interleukin-1β (IL-1β), interleukin-6 (IL-6), and tumor necrosis factor-α (TNF-α). JFP-Ps decreased inflammatory responses by inhibiting the production of gene and protein expression associated with the MAPK/ERK pathway. Additionally, metabolomic profiling revealed that LPS stimulation increased ether lipid metabolism while it decreased pantothenate and coenzyme A biosynthesis. These metabolic changes were partially reversed by JFP-Ps through inhibiting the synthesis of branched-chain amino acids. Conclusions: JFP-Ps may exert anti-inflammatory effects by concurrently modulating oxidative stress, inflammatory signaling, and metabolic reprogramming in macrophages.

## 1. Introduction

Inflammation serves as a crucial host defense mechanism in combating microbial infections, tissue damage, and environmental insults. An accumulating body of evidence indicating that there is a close relationship between chronic inflammation and various diseases, including autoimmune disorders, cancer, and neurological conditions [1,2]. Macrophages constitute a vital cellular element of the innate immune system and are widely distributed in tissues such as liver, intestine, and lung tissue. Lipopolysaccharide (LPS), a well-characterized macrophage activator, is frequently used to induce inflammatory responses in vitro [3]. In response to an LPS challenge, macrophages mainly generate cytokines that promote inflammation via stimulating the signaling pathways of mitogen-activated protein kinases (MAPKs) and nuclear factor-kappa B [4]. Thus, an inflammatory cellular model can be established using an LPS-activated RAW264.7 macrophage, a framework commonly used to evaluate the anti-inflammatory activity of dietary components and clarify their mechanistic pathways.

Using natural products as a source of anti-inflammatory compounds has grown in popularity in the past few decades. Scientists have dedicated a great deal of attention to polysaccharides derived from natural plants, particularly because of their anti-inflammatory qualities. Numerous reports showed that bioactive polysaccharides from natural plants exhibit anti-inflammatory effect might be related to MAPKs signaling pathway, which plays an important role in aspects of immune mediated inflammatory responses. For example, *Lycium barbarum* polysaccharides attenuated the LPS-induced inflammatory cascade in RAW264.7 cells by blocking Toll-like receptor (TLR)-4 signal transduction [5]. *Xanthii fructus* reduced pro-inflammatory responses by upregulating heme oxygenase-1 (HO-1) production and decreasing JNK/p38 MAPK phosphorylation in LPS-triggered macrophages [6]. Similarly, polysaccharides isolated from *Sparassis crispa* and *Astragalus* exhibited anti-inflammatory effects by modulating TLR-mediated MAPK pathway signaling [7,8]. *Phellinus linteus* polysaccharides had an inhibitory impact on the production of anti-inflammatory cytokines, such as TNF-α and IL-1β in LPS-primed RAW264.7 cells [9]. Polysaccharides derived from *Thuja occidentalis* Linn. exhibited the capacity to decrease oxidative stress levels and prevent the production of inflammation-associated cytokines (TNF-α and IL-6) [10]. Additionally, fractionation of polysaccharides from *Suaeda maritima* exerted a remarkable reduction in the secretion of inflammatory mediators including NO, inducible nitric oxide synthase (iNOS), cyclooxygenase-2 (COX-2); inhibited pro-inflammatory cytokines including TNF-α, IL-1β and IL-6; and blocked MAPK activation [11].

*Artocarpus heterophyllus* Lam. (jackfruit) is widely distributed in Asia and rich in carbohydrates (54.39%), protein (18.36%), and minerals [12]. Our previous work revealed that the polysaccharides derived from jackfruit pulp (JFP-Ps) are composed of five neutral sugars (rhamnose, arabinose, galactose, glucose, and xylose) and one uronic acid (galacturonic acid) and have potential bioactivities, such as in vitro anti-oxidation and in vivo immune-regulatory activities [13,14]. However, the anti-inflammatory effects of JFP-Ps on the cytokine production and MAPK activation remain insufficiently understood. We hypothesized that JFP-Ps could attenuate LPS-induced inflammation in macrophages by modulating oxidative stress, suppressing key inflammatory cytokines and blocking the activation of related signaling pathways. Therefore, our objective was to determine whether JFP-Ps mitigate LPS-induced inflammation in RAW264.7 macrophages and to delineate the involvement of MAPK/ERK pathway, complemented by non-targeted metabolomics via establishing LPS-activated RAW264.7 macrophages.

## 2. Materials and Methods

### 2.1. Materials and Chemicals

*Artocarpus heterophyllus* Lam. fruit was obtained from Spice and Beverage Research Institute, Chinese Academy of Tropical Agricultural Sciences (CATAS), Xinglong city, Hainan Province, China (18°4′ N, 110°1′ E). Crude polysaccharides were extracted from *Artocarpus heterophyllus* Lam. pulp using hot water and alcohol precipitation method, and removed protein by sevage method. Then JFP-Ps were purified using a Sephacryl™ S-400 HR column (1.6 × 60 cm, Sigma, St. Louis, MO, USA), which contained 79.12% total sugar and 15.65% uronic acid, and the purity was above 89.58%, while the molecular weight was estimated to be 1668 kDa. High-performance anion exchange chromatography with pulsed amperometric detection (HPAEC-PAD) chromatogram results showed that the JFP-Ps were composed of rhamnose, arabinose, galactose, glucose, xylose, and galacturonic acid. The JFP-Ps were freeze-dried and stored at −80 °C for further use [13].

Wuhan Pricella Biotechnology Co., Ltd. (Wuhan, China) provided fetal bovine serum (FBS) and Dulbecco’s modified Eagle’s medium (DMEM). Sigma-Aldrich Co., Ltd. (St. Louis, MO, USA) provided FITC-dextran and lipopolysaccharide (LPS). Grace Biochemistry Co., Ltd. (Suzhou, China) provided assay kits for catalase (CAT), superoxide dismutase (SOD), glutathione peroxidase (GSH-Px), and total protein. Shanghai Enzyme-linked Biotechnology Co., Ltd. (Shanghai, China) supplied the ELISA kits for cytokines associated with IL-6, TNF-α, and IL-1β. Proteintech (Wuhan, China) provided primary antibodies: JNK, phosphorylated-JNK (p-JNK), P38, phosphorylated-P38 (p-P38), ERK, phosphorylated-ERK (p-ERK) and β-actin. Beyotime Biotechnology (Shanghai, China) provided the BeyoECL Moon chemiluminescence kit and secondary antibodies. The other reagents used were analytically pure.

### 2.2. Cell Culture

The RAW264.7 macrophages were supplied by Wuhan Pricella Biotechnology Co., Ltd. (Wuhan, China). Cells were cultured at 37 °C in high-glucose DMEM supplemented with 10% FBS, 100 U/mL of the antibiotic penicillin, and 100 μg/mL of streptomycin in an incubator that was humidified with 5% CO_2_. After being cultivated in 6-well plates, the cells were further separated into five experimental groups: an LPS treatment group that was administered 1 mL of DMEM supplemented with 1 µg/mL of LPS, the control group was administered 1 mL of DMEM. For the LPS + JFP-Ps groups, cells were cultivated in media containing JFP-Ps at final concentrations of 40, 80, and 160 µg/mL for 2 h and then treated with LPS at a concentration of 1 µg/mL for an additional 22 h. Six replicates were used in each group for experiments.

### 2.3. Analysis of Cell Viability

To assess cell viability using the Cell Counting Kit-8 (CCK-8, Beyotime Biotechnology, Shanghai, China) assay, we seeded the cells onto a 96-wells plate after increasing their population density to 1.5 × 10^5^ cells/mL. After adherence, 100 μL of DMEM containing JFP-Ps at doses of 0, 20, 40, 80, 160, 320 and 640 μg/mL were administered. Absorbance was determined at 450 nm using a Synergy™ H1 microplate reader (BioTek, Winooski, VT, USA) after a 24-h incubation period and the addition of 10 μL of CCK-8 reagent. The viability of cells was subsequently assessed relative to the untreated control group.

### 2.4. Determination of Nitric Oxide (NO) Levels

In accordance with a previously established protocol, nitrite accumulation in culture supernatants was assessed to evaluate NO production [15]. Briefly, RAW 264.7 cells (1 × 10^5^ cells/well) were pre-incubated at 37 °C for 24 h, then treated with for 24 h as Section 2.2. The cell culture supernatant was collected, and NO levels were measured according to the manufacturer protocol of Griess Reagent kit (S0021S, Beyotime Biotechnology, Shanghai, China). Several dilutions of sodium nitrite (NaNO_2_) standards (0–100 μM) were used to generate a standard curve. A SynergyTM H1 plate reader (BioTek, Winooski, VT, USA) was then used to measure the wavelength at 540 nm, and NO levels were determined utilizing the standard curve.

### 2.5. Determination of Intracellular Reactive Oxygen Species (ROS)

ROS levels were determined according to a previous report [16] and using an ROS assay kit (S0033S, Beyotime Biotechnology, Shanghai, China) according to the manufacturer’s protocol. Briefly, after incubation for 24 h, the cells were collected by removing the culture medium. 2,7-dichloro-dihydro-fluorescein diacetate diluted 1:100 in a culture medium (10 µmol/L) was added to the collected cells. Then the supernatant was aspirated after incubation at 37 °C for 20 min, the cells were gently rinsed three times with pre-warmed phosphate-buffered saline (PBS) to completely remove the 2,7-dichloro-dihydro-fluorescein diacetate. A Synergy^TM^ H1 microplate scanner (BioTek, Winooski, VT, USA), using a wavelength for stimulation of 488 nm and an emission wavelength of 525 nm, was used to measure fluorescence intensities. ROS levels were expressed as fold change relative to the untreated control group.

### 2.6. Measurement of Apoptosis

Apoptosis was determined utilizing the Annexin V-FITC apoptosis detection kit (C1062M, Beyotime Biotechnology, Shanghai, China). Cells from various groups were collected after being cleaned with PBS and centrifuged at 300× *g* (5 min, 4 °C), and suspended in 195 μL of 1× Annexin V binding buffer (pH 7.4). Subsequently, 5 μL Annexin V-FITC and 10 μL propidium iodide (PI) were added and mixed completely. After incubation at 25 °C in the dark for 15 min, the signals were analyzed on a FV10i confocal fluorescent microscopy (Olympus, Tokyo, Japan) under 60× magnification.

### 2.7. Assay of Antioxidant Activities

The cells were lysed in 1 mL of air-equilibrated Tris-HCl buffer (45 mmol/L, pH 7.87) containing 1 mmol/L EDTA after being incubated for 24 h and then washed three times with PBS. Following the kit’s instructions, we subsequently acquired the cell lysates in order to evaluate CAT, SOD, and GSH-Px activity. The protein amounts of cell lysates were measured using the bicinchoninic acid (BCA) assay kit (Beijing Biomed Gene technology, Beijing, China). Absorbance was measured using a Synergy^TM^ H1 microplate scanner (BioTek, Winooski, VT, USA).

### 2.8. Cytokine Assay

Culture supernatants were harvested using ELISA kits (Shanghai Enzyme-linked Biotechnology Co., Ltd., Shanghai, China) according to the manufacturer’s instructions to assess the levels of TNF-α, IL-6, and IL-1β.

### 2.9. Real Time Quantitative PCR

TRIzol reagent was used to separate total RNA from RAW264.7 macrophages (Beijing Solarbio Science and Technology Ltd., Beijing, China). A BeyoRT™ III first-strand cDNA synthesis kit (Beyotime Institute of Biotechnology, Shanghai, China) was synthesized using the first-strand cDNA from these RNA samples. We carried out real-time quantitative PCR (RT-qPCR) using Green qPCR MasterMix (High ROX) (Beijing Biomed Gene technology, Beijing, China), β-actin was used as the underlying standard gene for establishing the normal the expression of genes, and the gene-specific primers used are listed below:

β-actin (5′-3′) CTGAGAGGGAAATCGTGCGTGAC, (3′-5′) AGGAAGAGGATGCGGCAGTGG
IL-6 (5′-3′) CTTCTTGGGACTGATGCTGGTGAC, (3′-5′) TCTGTTGGGAGTGGTATCCTCTGTG
TNF-α (5′-3′) GGACTAGCCAGGAGGGAGAACAG, (3′-5′) GCCAGTGAGTGAAAGGGACAGAAC
IL-1β (5′-3′) AATCTCACAGCAGCATCTCGACAAG, (3′-5′) TCCACGGGCAAGACATAGGTAGC

### 2.10. Western Blotting

RAW264.7 macrophages were lysed in RIPA buffer containing PMSF/protease inhibitors for 30 min on ice. Supernatants were gathered after centrifugation (12,000× *g*, 4 °C, 5 min). Protein amounts were measured using the BCA assay kit (Beijing Biomed Gene technology, Beijing, China). After being denatured for five minutes at 95 °C, comparable amounts of protein (30 μg/lane) were separated using 10% SDS-PAGE and then put onto membranes made of PVDF (Millipore, Billerica, MA, USA) under 300 mA for 90 min. After blocking the transferred PVDF membranes with blocking buffer (5% dry milk, 0.1% Tween-20 in 1× Tris-buffered saline). The primary antibodies of JNK, p-JNK, ERK, p-ERK, p38, p-P38 and β-actin were used and incubated for an entire night at 4 °C. The membranes were washed three times with 1× Tris-buffered saline with Tween-20 after a 12-h incubation, then treated with secondary antibodies for additional 2 h at 4 °C. BeyoECL Plus (Beyotime Biotechnology, Shanghai, China) was used to observe the bands via the Tanon 5200 Imaging System (Tanon, Shanghai, China). β-actin was used to normalize data and the ImageJ 1.8.0 software was used for analysis.

### 2.11. Analysis of UPLC-Q-TOF-MS/MS

A modified procedure was used to determine metabolites from RAW264.7 macrophage cells [17]. The culture supernatant was combined with methanol and acetonitrile in a 20:40:40 (*v*/*v*/*v*) ratio; the mixture was then swirled for 30 s and centrifuged at 12,000× *g* for 5 min at 4 °C in order to extract extracellular metabolites. The supernatant was collected for UPLC-Q-TOF-MS and UPLC-Q-TOF-MS/MS examination after being passed through a 0.22 μm filter. Moreover, cells were collected and extracted using a 40:40:20 (*v*/*v*/*v*) mixture of methanol, acetonitrile, and water, along with ultra-sonicated for 30 min at 4 °C, then centrifuged at 12,000× *g* for 20 min. The supernatants were collected, filtered using 0.22 μm membranes, and transferred to HPLC vials. 3 μL quantities of samples were injected into the UHPLC system at a flow rate of 0.4 mL/min equipped with an Agilent ZORBAX RRHD Eclipse Plus C18 column (3.0 mm × 150 mm, 1.8 μm, Agilent Technologies, Santa Clara, CA, USA). Methanol (phase B) and 0.1% liquid formic acid (phase A) made up the mobile phases. Data on mass spectroscopy were gathered with an Agilent 6530B Q-TOF-MS/MS (Agilent Technologies, Santa Clara, CA, USA).

The raw UPLC-Q-TOF-MS data were acquired using the MassHunter Workstation Software (version B.06.01, Agilent Technologies, Santa Clara, CA, USA). Alignment, normalization, defining the sample sets, filtering by frequency and Venn diagram analysis were processed using the Mass Profiler Professional (version B.14.0, Agilent Technologies, Santa Clara, CA, USA). Principal component analysis (PCA) was employed to differentiate metabolic profiles among the various experimental groups. To find metabolites that were distinctive, a fold change (FC) > 2 and *p* < 0.05 were utilized, and their identities were annotated by database matching and further validated via UPLC-Q-TOF-MS/MS.

### 2.12. Statistical Analysis

The data was presented as means ± standard deviations (SDs). One-way ANOVA followed by Tukey’s post hoc test was used for statistical evaluations via SPSS (version 16.0, Chicago, IL, USA, SPSS Inc.) and drawn using the GraphPad Prism 6.0 (San Diego, CA, USA). Differences were considered statistically significant at *p* < 0.05 or *p* < 0.01.

## 3. Results

### 3.1. Cell Viability, NO Production, and Intracellular ROS Levels

**Figure 1:**
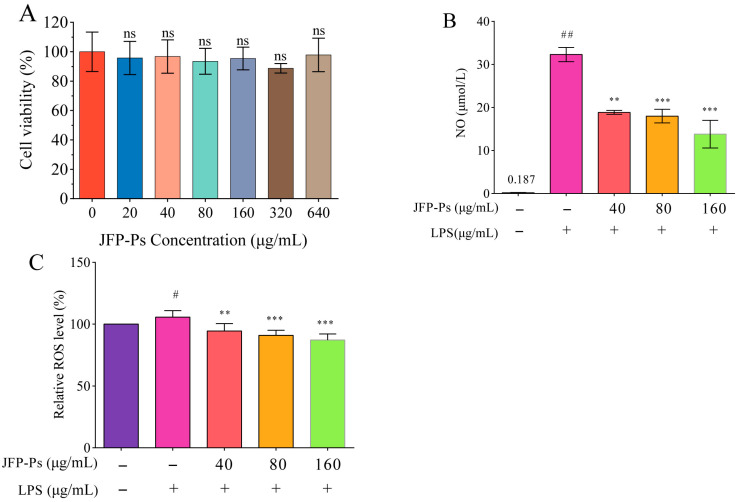
Effects of JFP-Ps on cell viability (**A**), NO production (**B**), and ROS production (**C**) in LPS-treated RAW264.7 cells. # *p* < 0.05 and ## *p* < 0.01 compared with the control group, and ** *p* < 0.01 and *** *p* < 0.001 compared with the LPS-treated group (*n* = 6). “ns” means no significant difference.

No significant changes in cell viability were observed at JFP-Ps concentrations of 0, 20, 40, 80, 160,320 and 640 μg/mL (Figure 1A), suggesting that JFP-Ps did not exert cytotoxic effects within this concentration range. Therefore, JFP-Ps at concentrations of 40, 80, and 160 μg/mL were selected for further experiments.

As demonstrated in Figure 1B, NO levels were significantly elevated to 32.31 μmol/L after LPS administration compared to that in the untreated group (0.187 μmol/L). However, JFP-Ps effectively suppressed NO generation, exhibiting a concentration-dependent response at a significant level (*p* < 0.05).

Meanwhile, LPS stimulation significantly increased ROS production in RAW264.7 cells, whereas JFP-Ps administration efficiently decreased intracellular ROS levels relative to the LPS group (*p* < 0.01) (Figure 1C). It was considered that LPS could be used as an inflammatory stimulus and JFP-Ps has potential capability to inhibit LPS-induced NO and ROS production in Raw264.7 cells.

### 3.2. Impact of JFP-Ps on Apoptosis

**Figure 2:**
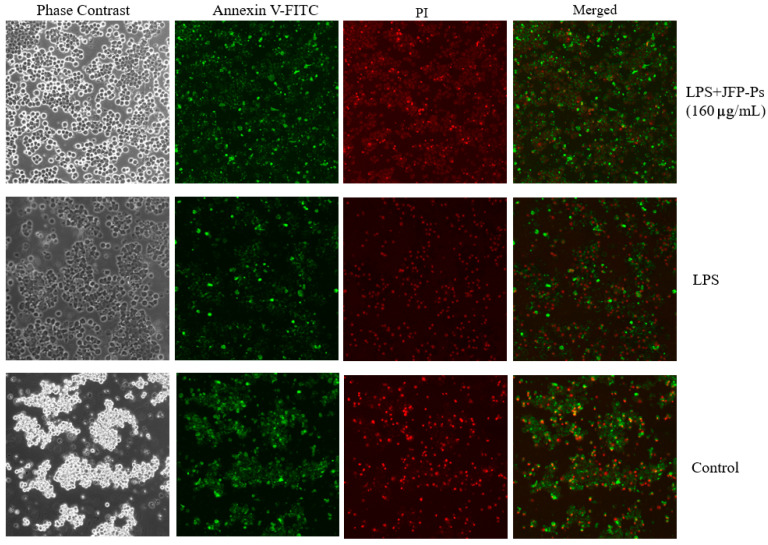
Apoptosis triggered by LPS and LPS+JFP-Ps (160 μg/mL) in RAW264.7 cells. Note: The effect of Annexin V-FITC and propidium iodide (PI) staining. The green fluorescence corresponds to Annexin V-FITC-staining-positive cells, and the red fluorescence corresponds to propidium-iodide-staining-positive cells. Apoptotic cells were only stained with green fluorescent dye, necrotic cells were double stained with green and red fluorescent dyes, and normal cells were not stained with fluorescent dye. LPS induced the occurrence of apoptosis in Raw264.7 cells, while 160 μg/mL JFP-Ps effectively mitigated LPS-induced apoptosis.

As demonstrated in Figure 2, after exposure to LPS treatment alone, the RAW264.7 cells exhibited nuclear condensation and annexin V/PI positivity, indicating the occurrence of apoptosis. The administration of LPS increased red fluorescence (corresponding to apoptotic/necrotic cells) and decreased green fluorescence (corresponding to viable cells) in comparison to the control group. However, the LPS+JFP-Ps group exhibited increased green fluorescence and reduced red fluorescence, suggesting that JFP-Ps at the concentration of 160 μg/mL effectively mitigated LPS-induced apoptosis.

### 3.3. Effect of JFP-Ps on the Activity of Antioxidant Enzymes

As demonstrated in Table 1, CAT activity was significantly increased after LPS exposure, consistent with the oxidative stress state induced by LPS. But CAT activity was significantly diminished (*p* < 0.05) by JFP-Ps at various concentrations. LPS substantially lowered the production of antioxidant enzymes including SOD and GSH-Px. However, JFP-Ps significantly reversed the activities of the two enzymes. These findings indicated that JFP-Ps might enhance the production of GSH-Px and SOD and protect RAW264.7 cells against LPS-induced damage caused by oxidative stress.

### 3.4. Inhibition of Pro-Inflammatory Cytokine Production via JFP-Ps

**Figure 3:**
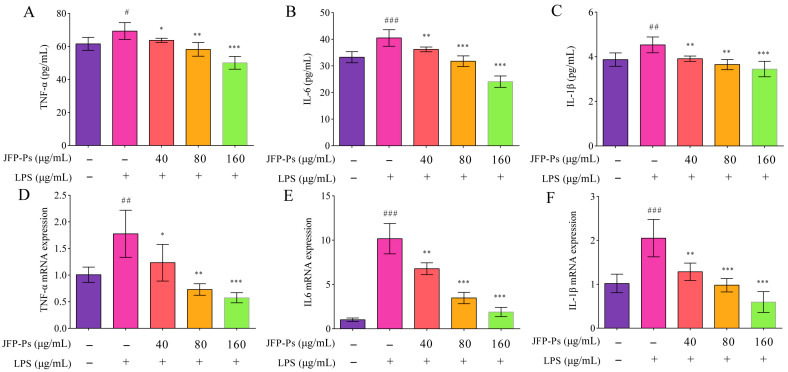
Inhibitory effect of JFP-Ps on pro-inflammatory cytokines (TNF-α, IL-6, and IL-1β) and their mRNA expression in LPS-stimulated RAW264.7 cells. Pro-inflammatory cytokines: TNF-α (**A**), IL-6 (**B**), and IL-1β (**C**). mRNA expression: TNF-α (**D**), IL-6 (**E**), and IL-1β (**F**). # *p* < 0.05, ## *p* < 0.01, and ### *p* < 0.001 compared with the control group, and * *p* < 0.05, ** *p* < 0.01, and *** *p* < 0.001 compared with the LPS-treated group.

The production and mRNA expression of IL-1β, TNF-α, and IL-6 were markedly elevated (*p* < 0.01) in the LPS-stimulated RAW264.7 cells (Figure 3A–C), which promoted inflammation. However, JFP-Ps reduced the production of these inflammatory cytokines in a dose-responsive way (*p* < 0.01). Additionally, JFP-Ps treatment led to a significant downregulation of the mRNA expression of IL-1β TNF-α, and IL-6 (*p* < 0.05), and this regulation was dose-dependent (Figure 3D–F). These findings implied that JFP-Ps might exert an anti-inflammatory effect by suppressing pro-inflammatory cytokines’ transcriptional levels and lowering their release.

### 3.5. Impact of JFP-Ps on MAPK Signaling Pathway

**Figure 4:**
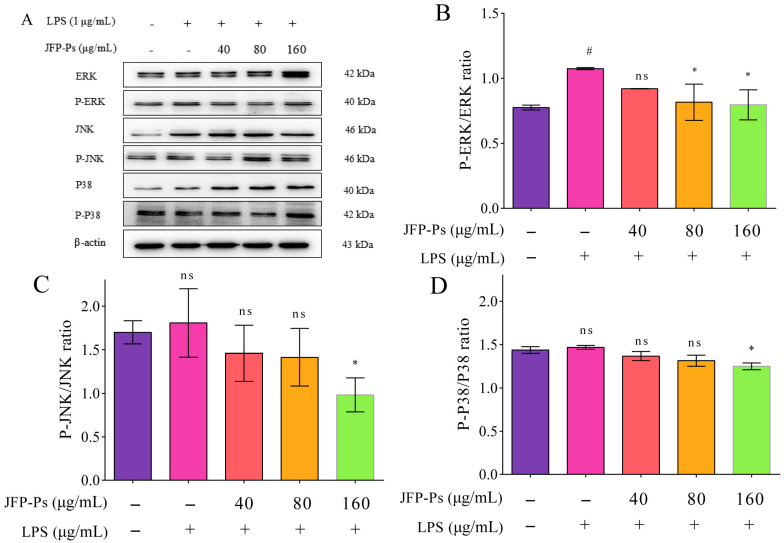
The effect of JFP-Ps on the expression of the MAPK/ERK signaling pathways in LPS-stimulated RAW 264.7 cells. (**A**)—protein expression; (**B**)—P-ERK/ERK; (**C**)—P-JNK/JNK; (**D**)—P-P38/P38. # *p* < 0.05 compared with the control group. * *p* < 0.05 compared with the LPS-treated group. “ns” means no significant difference.

As shown in Figure 4, the Western blotting results indicated that LPS stimulation increased ERK, JNK, and P38 phosphorylation, with the results for ERK phosphorylation being statistically significant. This finding suggests that ERK occupies a key position in the inflammatory reaction triggered by LPS. JFP-Ps markedly reduced ERK phosphorylation (*p* < 0.05), indicating that JFP-Ps primarily suppresses LPS-induced inflammation through ERK inhibition, as well as exerts inhibition effects on JNK and P38.

### 3.6. Non-Targeted Metabolomic Analysis of JFP-Ps in LPS-Induced RAW264.7 Cells

**Figure 5:**
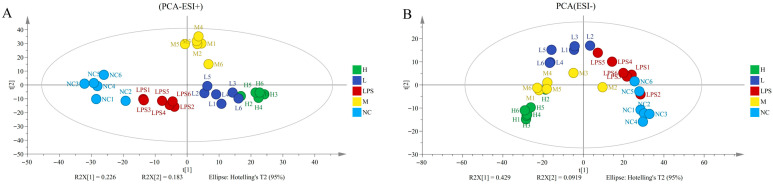

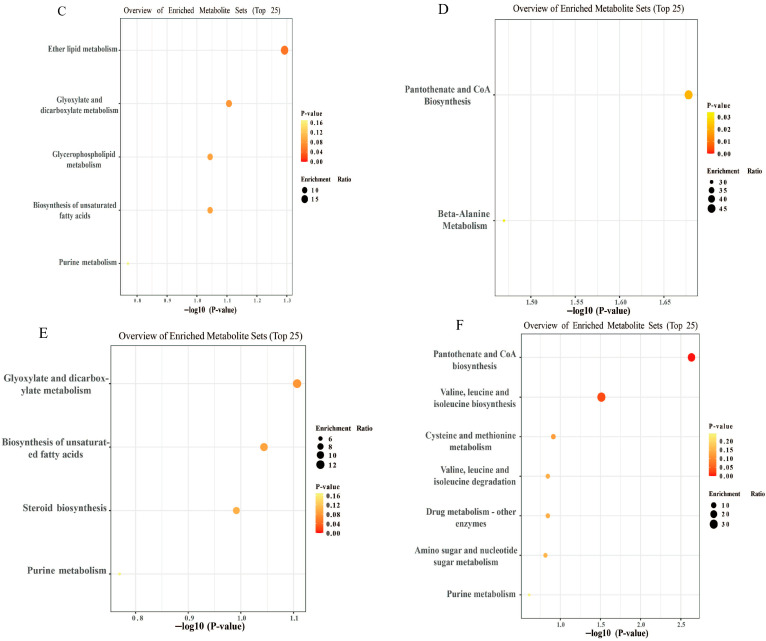
Metabolomic analysis of JFP-Ps in LPS-stimulated RAW 264.7 cell intracellular samples. (**A**)—PCA score plot of positive ion mode; (**B**)—PCA score plot of negative ion mode; (**C**)—the upregulated enrichment pathway in the LPS group vs. that in the NC group; (**D**)—the downregulated enrichment pathway in the LPS group vs. that in the NC group; (**E**)—the upregulated enrichment pathway in the LPS group vs. that in the H group; (**F**)—the downregulated enrichment pathway in the LPS group vs. that in the H group. The metabolomic analysis results of JFP-Ps in LPS-stimulated RAW 264.7 cell intracellular samples from five groups were all separated in the PCA solution. Pathway enrichment analysis results of cell intracellular showed that JFP-Ps alleviated inflammation by modulating lipid metabolism and branched-chain amino acid pathways. NC—intracellular fluid of the control group; LPS—LPS group at 1 µg/mL; L—JFP-Ps low-dose group at 40 μg/mL of JFP-Ps; M—JFP-Ps medium-dose group at 80 μg/mL of JFP-Ps; H—JFP-Ps high-dose group at 160 μg/mL of JFP-Ps.

**Figure 6:**
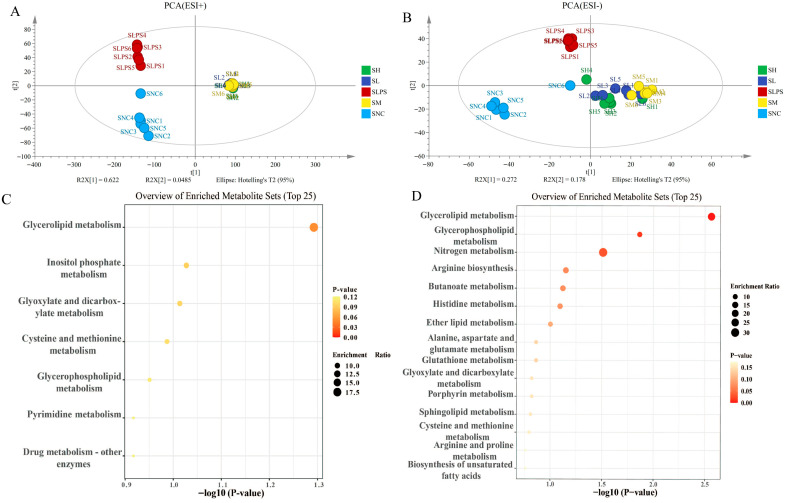
Metabolomic analysis of JFP-Ps on LPS-stimulated RAW 264.7 cell culture supernatant samples. (**A**)—PCA score plot of positive ion mode; (**B**)—PCA score plot of negative ion mode; (**C**)—the upregulated enrichment pathway in the SLPS group vs. that in the SNC group; (**D**)—the upregulated enrichment pathway in the SLPS group vs. that in the SH group. The metabolomic analysis results of JFP-Ps in LPS-stimulated RAW 264.7 cell culture supernatant samples from five groups were all separated in the PCA solution. Pathway enrichment analysis results of cell intracellular showed that JFP-Ps alleviated inflammation by modulating lipid remodeling, energy metabolism, and redox-related pathways. SNC—extracellular fluid of normal control group; SLPS—extracellular fluid of LPS group at 1 µg/mL; SL—extracellular fluid of JFP-Ps low-dose group at 40 μg/mL of JFP-Ps; SM—extracellular fluid of JFP-Ps medium-dose group at 80 μg/mL of JFP-Ps; SH—extracellular fluid of JFP-Ps high-dose group at 160 μg/mL of JFP-Ps.

Non-targeted metabolomic testing was conducted to further explore how JFP-Ps regulate LPS-induced metabolic disruptions. The NC, LPS, and JFP-Ps groups were clearly separated via principal component analysis (PCA) in cell intracellular samples (Figure 5A,B) and cell culture supernatant samples (Figure 6A,B). This finding implied that cellular metabolism was significantly disrupted by LPS, but these metabolic alterations were partially reversed and returned to normal by the JFP-Ps.

After completing this, differentiation, significance testing and fold change analysis, and Principal Component Analysis (PCA) were performed in version B.14.0 Mass Profiler Professional software. 83 significantly altered metabolites were identified between the NC and LPS groups (42 upregulated and 41 downregulated) and 180 between the LPS and high-dose-JFP-Ps group (94 upregulated and 86 downregulated) in cell intracellular samples. 444 significantly altered metabolites were identified between the NC and LPS groups (332 upregulated and 112 downregulated) and 1760 between the LPS and high-dose-JFP-Ps group (1700 upregulated and 60 downregulated) in cell culture supernatant samples.

Pathway enrichment analysis of cell intracellular samples showed that LPS stimulation markedly upregulated ether lipid metabolism, glyoxylate and dicarboxylate metabolism, glycerophospholipid metabolism, and unsaturated fatty acid biosynthesis (Figure 5C), while it downregulated pantothenate and CoA biosynthesis and β-alanine metabolism (Figure 5D). However, high-dose of JFP-Ps upregulated glyoxylate and dicarboxylate metabolism and unsaturated fatty acid biosynthesis (Figure 5E), while notably downregulated valine, leucine, and isoleucine biosynthesis (Figure 5F), pathways often associated with inflammatory activation. These results implied that JFP-Ps alleviated metabolic dysregulation by modulating lipid metabolism and branched-chain amino acid pathways.

Similarly, pathway enrichment analysis of the culture supernatant showed that LPS altered multiple pathways related to amino acids and lipid metabolism, as well as the oxidative stress response (Figure 6C). JFP-Ps significantly restored pathways such as glycerolipid metabolism, glycerophospholipid metabolism, and glutathione metabolism (Figure 6D), indicating enhanced antioxidant capacity and improved metabolic homeostasis. These findings demonstrated that JFP-Ps mitigated LPS-induced metabolic reprogramming in RAW264.7 cells, particularly by modulating lipid remodeling, energy metabolism, and redox-related pathways, thereby contributing to its anti-inflammatory effect.

## 4. Discussion

Jackfruit extract has been reported to exhibit anti-diabetic and antioxidant activities, which are associated with a reduction in ROS production [18]. Similarly, JFP-Ps has been reported to exhibit antioxidant properties in vitro and implicated in modulating obesity by altering intestinal microbiota composition [13,19]. Importantly, JFP-Ps markedly reduced the quantity of ROS in LPS-stimulated RAW264.7 cells, thereby confirming its antioxidant capabilities.

LPS-induced oxidative stress and inflammation are mainly brought about by excessive production of ROS, compromising the defense against antioxidants mechanisms, ultimately resulting in a number of pathophysiological consequences [20,21]. Catalase (CAT), superoxide dismutase (SOD), and glutathione peroxidase (GSH-Px) are key antioxidant enzymes maintaining cellular redox homeostasis [22]. Consistent with earlier research findings, this investigation demonstrates that LPS stimulation significantly raises ROS levels, markedly increasing CAT activity inside RAW264.7 macrophages. Notably, JFP-Ps substantially decreased ROS levels and significantly reduced CAT activity dose-dependently in LPS-treated RAW264.7 cells, consistent with a report on polysaccharides extracted from *Tetrastigma hemsleyanum* roots [23]. Furthermore, GSH-Px and SOD activities were significantly suppressed by LPS but restored by JFP-Ps. This finding suggested that JFP-Ps enhanced cellular antioxidant defenses against oxidative stress, a finding corroborated by studies on polysaccharides from *Porphyra haitanensis* and *Dendrobium devonianum* [24].

It has been demonstrated that jackfruit extracts containing ethyl acetate prevent the synthesis of ROS, prostaglandin E2 (PGE2), and NO generation in RAW264.7 cells stimulated by LPS, suggesting that jackfruit has anti-inflammatory properties [25]. In our study, JFP-Ps treatment decreased NO production concentration-dependently, indicating effective suppression of LPS-induced inflammatory activation. Capable of reacting to a range of stimuli, macrophages are vital components of the innate immune system and can release cytokines that are associated with inflammation [26]. According to this study, LPS markedly increased macrophages’ pro-inflammatory cytokine production. Nevertheless, JFP-Ps treatment caused these quantities of cytokines to drop significantly in a dose-dependent manner. Parallel reductions were observed at the mRNA-expression level, confirming transcriptional suppression of pro-inflammatory genes. These results were consistent with other studies showing that Astragalus-derived polysaccharides, okra, and honey-processed *Astragalus* exhibited anti-inflammatory activities by inhibiting similar cytokines [27,28,29]. The overall results of this investigation showed that JFP-Ps inhibited inflammatory responses through effectively reducing the synthesis and transcription of key pro-inflammatory mediators.

Multiple signaling pathways are engaged in the inflammatory reaction of RAW264.7 cells, and members of the MAPK family serve as crucial participants in cell proliferation, differentiation, and stress responses [30]. LPS stimulation significantly increased ERK phosphorylation, but JFP-Ps treatment markedly inhibited ERK phosphorylation, indicating that JFP-Ps suppressed inflammatory signaling primarily through targeting ERK. However, JNK and P38 phosphorylation were not significantly affected, suggesting that JFP-Ps selectively modulated the MAPK pathway. Similar anti-inflammatory effects have been reported for polysaccharides derived from *Ginkgo biloba exocarp* and *Phellinus igniarius* [31,32]. Collectively, JFP-Ps inhibited inflammatory responses via dual modulation of the MAPK (mainly ERK) pathway, providing an experimental basis for understanding the anti-inflammatory mechanism in macrophages.

A UPLC-Q-TOF-MS-based metabolism study was performed to better understand the role of JFP-Ps in LPS-induced metabolism dysregulation. The administration of LPS resulted in a substantial disruption of lipid, energy, and amino acid metabolism. LPS markedly upregulated ether lipid metabolism, glycerophospholipid metabolism, and unsaturated fatty acid biosynthesis, with ether lipid metabolism showing the most significant activation. Previous studies have demonstrated that LPS enhanced ether lipid synthesis by promoting the generation of pro-inflammatory lipid mediators that further amplify MAPK pathway activation [33]. Similar metabolic disturbances have been reported with respect to *Flammulina velutipes* polysaccharide interventions, highlighting the central role of lipid remodeling [34]. In contrast, LPS substantially impeded the synthesis of pantothenate and CoA as well as the metabolism of β-alanine. The tricarboxylic acid (TCA) cycle and fatty acid oxidation both depend on pantothenic acid (vitamin B5), which is a precursor of these acids [35]. Impairment of these pathways suggests reduced ATP generation and mitochondrial dysfunction. Moreover, β-alanine metabolism is closely linked to CoA biosynthesis [36], and its downregulation could exacerbate energy deficits [37].

Amino acid metabolism also plays a key role in inflammatory regulation. Branching-chain amino acid (BCAA) synthesis was markedly inhibited by JFP-Ps treatment. Inhibition of BCAA biosynthesis can modulate inflammatory and metabolic pathways, including cAMP signaling [38,39]. Moreover, the decomposition products produced by BCAAs play an essential role in controlling oxidative stress in the mitochondria, and JFP polysaccharides may indirectly affect inflammation by modulating these metabolic pathways [14,40]. When combined, our results show that JFP-Ps reduced inflammation brought on by LPS via modifying lipid metabolism, restoring mitochondrial energy pathways, and regulating amino acid biosynthesis.

## 5. Conclusions

This study evaluated the anti-inflammatory effects of JFP-Ps on cytokine production and MAPK activation in LPS-stimulated RAW264.7 macrophages. The results revealed that JFP-Ps decreased the production of TNF-α, IL-1β, IL-6, and NO in RAW264.7 macrophages activated by LPS. JFP-Ps primarily suppressed LPS-induced inflammation through ERK, JNK and P38 inhibition. Metabolomic results showed that JFP-Ps reduced inflammation by modifying lipid metabolism, restoring mitochondrial energy pathways, and regulating amino acid biosynthesis. However, due to the complexity of polysaccharide components, future studies should explore structure–function relationships and validate these findings in in vivo models.

## Figures and Tables

**Table 1:** Effects of JFP-Ps on antioxidant enzyme activity in LPS-RAW264.7 cells.

	Control Group	LPS	LPS+JFP-Ps Concentrations (μg/mL)		
			40	80	160
CAT (µmol/min/mg prot)	59.91 ± 10.05 ^ab^	69.18 ± 13.46 ^a^	53.98 ± 3.09 ^bc^	46.39 ± 2.32 ^cd^	37.49 ± 8.03 ^d^
GSH-Px (µmol/mg prot)	253.51 ± 23.23 ^a^	168.37 ± 13.82 ^b^	191.11 ± 11.93 ^b^	224.52 ± 31.33 ^a^	257.32 ± 29.49 ^a^
SOD (U/mg prot)	0.54 ± 0.064 ^a^	0.29 ± 0.038 ^c^	0.42 ± 0.042 ^b^	0.46 ± 0.041 ^b^	0.52 ± 0.032 ^a^

CAT—catalase; GSH-Px—glutathione peroxidase; SOD—superoxide dismutase. Data are expressed as means ± SDs (*n* = 6). Values with the different letters in the same column are significantly different (*p* < 0.05).

## Data Availability

The data presented in this study are available from the corresponding author on reasonable request due to technical/time limitations.

## Abbreviations

JFP-Ps, polysaccharide from *Artocarpus heterophyllus* Lam.; LPS, lipopolysaccharide; NO, nitric oxide; ROS, reactive oxygen species; GSH-Px, glutathione peroxidase; SOD, superoxide dismutase; CAT, catalase; IL-1β, interleukin-1β; IL-6, interleukin-6; TNF-α, tumor necrosis factor-α; MAPKs, mitogen-activated protein kinases; TLR, Toll-like receptor; HO-1, heme oxygenase-1; iNOS, inducible nitric oxide synthase; COX-2, cyclooxygenase-2; HPAEC-PAD, High-performance anion exchange chromatography with pulsed amperometric detection; FBS, fetal bovine serum; DMEM, Dulbecco’s modified Eagle’s medium; CCK-8, Cell Counting Kit-8; BCA, bicinchoninic acid; PGE2, prostaglandin E2; TCA, tricarboxylic acid.
